# Prevalence and management of immediate drug hypersensitivity observed during antineoplastic drug therapy

**DOI:** 10.2340/1651-226X.2025.43098

**Published:** 2025-04-28

**Authors:** Trine Holm Rasmussen, Charlotte Gotthard Mortz, Per Pfeiffer, Nina Andersen, Carsten Bindslev-Jensen

**Affiliations:** a Department of Dermatology and Allergy Center, Odense Research Centre for Anaphylaxis (ORCA), Odense University Hospital, Odense, Denmark; b Department of Oncology, Odense University Hospital, Odense, Denmark; c Department of Hematology, Odense University Hospital, Odense, Denmark

**Keywords:** Drug allergy, infusion reaction, chemotherapy, biologics, checkpoint inhibitors, rechallenge, premedication

## Abstract

**Background:**

Immediate drug hypersensitivity reactions (IDHRs) complicate the treatment of patients with cancer. Rapid drug desensitization (RDD) is not a standard treatment option in Northern Europe as in Southern Europe and the US. Thus, in Denmark, allergists are not involved when cancer treatments are complicated by IDHRs.

**Purpose:**

The purpose was to investigate whether Danish patients could benefit from the implementation of an allergy work-up including RDD by investigating the magnitude of the problem with IDHRs in Danish antineoplastic drug therapy, in addition to describe characteristics of IDHRs, re-treatment strategies, and outcomes.

**Patients and methods:**

This prospective observational single-center study was conducted at a large university hospital. Patients were included over 17 months. Patients were interviewed during index reaction. Information on culprit drug, infusion procedure, and premedication was obtained, together with reaction phenotype and severity. After 3 months, information on re-treatment strategies and outcome were obtained from medical records.

**Results:**

In total, 126 patients experienced IDHRs during the study period. This corresponds to 2.5% of patients receiving antineoplastic drug therapy. Re-treatment, using increased premedication and/or decreased infusion rate, was attempted in 97 patients and tolerated by 69. However, 57 out of 126 patients (45%) discontinued treatment. This corresponds to 1.1% of patients receiving antineoplastic drug therapy. Patients with gynecologic cancers had a particularly high risk.

**Interpretation:**

IDHRs are infrequent in antineoplastic drug therapy, but due to the large number of patients with cancer, the number of IDHRs is significant. Patients discontinuing treatment could benefit from an allergy work-up including RDD.

## Introduction

Antineoplastic drug therapy can cause immediate drug hypersensitivity reactions (IDHRs) complicating the treatment of patients with cancer [1, 2]. Previous studies have reported the prevalence of IDHRs for specific antineoplastic drugs [3], but to our knowledge, the overall prevalence of IDHRs and their consequences in patients with cancer has not previously been described.

In cancer treatment, taxanes, platinum salts, and biologics are the drug classes most often involved in IDHRs. For selected drugs, the reported prevalences of IDHRs are: paclitacel 2–10% [3, 4], carboplatin 2–12% [5], oxaliplatin 2–25% [6], rituximab 5–10%, cetuximab 3–22%, trastuzumab 1–5% [7], nivolumab 5–10% [8, 9], and ipilimumab 2–6% [10].

IDHRs vary in severity from mild reactions limited to the skin to life-threatening anaphylaxis. IDHRs include both allergic reactions and other reaction types such as cytokine-release reactions. The underlying mechanism of the IDHR cannot be determined from symptoms alone but may be indicated by the phenotype [11–13]. Identifying the reaction phenotype can help clinicians to manage the acute situation and plan future treatment.

Premedication is used in treatment with certain drugs to prevent IDHRs. Examples include treatment with taxanes and rituximab, where premedication with glucocorticoids and antihistamines has reduced the incidence of IDHRs [4, 5, 14, 15]. Additionally, decreased infusion rate can be used during first-time treatment to prevent IDHRs [15]. Increased premedication and decreased infusion rate can also be used when re-treating patients after IDHRs. However, re-treatment strategies used in cancer departments are not well described in literature. Re-treatment has been described as being generally successful with taxanes [4, 16, 17] and biologics [18], including checkpoint inhibitors [15, 19], but less successful with platinum salts [15, 17, 20, 21].

When antineoplastic treatments are not tolerated due to IDHRs, patients risk discontinuing critical antineoplastic treatment. To address this problem, allergy centers in Southern Europe and the US have been offering rapid drug desensitization (RDD) to patients for more than 15 years [1, 2]. RDD induces a transient tolerance to the culprit drug. The drug infusion is initiated with extremely low doses, which are gradually increased over time until the patient has received a full target dose within hours. The technique allows almost 100% of patients to continue treatment with the culprit drug [1, 2]. In Northern Europe, allergists are not routinely involved in the management of patient with IDHRs elicited by antineoplastic drugs, and RDD is not a treatment option.

The number and proportion of patients who are unable to continue treatment with an antineoplastic drug at the cancer department due to IDHRs are lacking in the literature. We therefore aimed to determine the extent to which IDHRs were a problem in Danish cancer treatment to see whether Danish patients potentially would benefit from an allergy work-up including RDD. To better identify patients in need of such a service, we also aimed to describe the clinical features of IDHRs, how patients were re-treated at the cancer department and the outcome of re-treatment.

The objectives of this study were: (1) to estimate the prevalence of IDHRs in antineoplastic drug therapy and (2) to estimate the prevalence of patients discontinuing treatment due to such reactions. Furthermore, (3) to describe reaction patterns related to drug classes, (4) to describe re-treatment strategies and (5) to describe the outcome of re-treatment.

## Methods

### Study design and study participants

This study was a prospective observational study. Patients were investigated at the time of first IDHR and reassessed 3 months later.

During the inclusion period from 01 August 2022 to 31 December 2023, all patients, aged 18 years or above, were included if they were treated at the outpatient infusion center at the Departments of Oncology and Hematology at Odense University Hospital and experienced an IDHRs elicited by antineoplastic drugs.

### Patient inclusion and factors related to the IDHR

Nurses at the outpatient infusion center immediately contacted the Allergy Center at Odense University Hospital when an IDHR occurred. Patients were contacted directly at bedside and examined and interviewed about the reaction. When an IDHR occurred after a patient was discharged, a phone interview or an interview was conducted at next visit.

Nurses at the outpatient infusion center provided information on timing, observed symptoms and vital values in patients with IDHRs. Information was obtained about premedication used, timing of drugs administered, and infusion rates. The medical record was accessed for information about cancer type and previous cancer treatments.

The study was observational. The authors did not interfere with the management of IDHRs nor the planning of subsequent antineoplastic treatments. These decisions were made by oncologists and hematologists.

### Re-treatment strategies and outcome

Medical records were assessed 3 months after the IDHR for information about further cancer treatment. Information was collected on how any re-treatment was conducted, including changes such as increased premedication and decreased infusion rate. It was recorded whether the re-treatment was successful, which was defined by the patient being able to receive full treatment. When re-treatments were unsuccessful, we obtained information about the new IDHR. If an antineoplastic treatment was discontinued, we recorded whether the reason was IDHRs or other causes.

### Calculation of prevalences

A report from the hospital pharmacy included all antineoplastic intravenous and subcutaneous formulations delivered to the outpatient infusion center at Odense University Hospital (OUH) during the study period. The number of unique patients receiving medical antineoplastic therapy in total, with a specific drug and within the different cancer types was recorded and used to calculate prevalences.

Chemotherapeutics were divided into three drug classes: platinum salts, taxanes, and *other* chemotherapeutics. Biologics were divided into two drug classes: checkpoint inhibitors and *other* biologics.

### Reaction phenotype and severity of IDHRs

An IDHR was defined as symptoms and signs of hypersensitivity appearing during or within 6 h after treatment. IDHRs were categorized into four reaction phenotypes based on observed symptoms: type-1, cytokine-release, mixed-type, and either-type. The phenotyping system has been suggested by a recognized research group [13].

Type-1 reactions include at least one symptom suggestive of mast cell/basophil degranulation: pruritus, urticaria, angioedema, nasal congestion, sneezing, wheezing, coughing, throat tightness, and tongue swelling. Nonspecific symptoms may be included [13].

Cytokine-release reactions include at least one symptom suggestive of cytokine release: chest pain, back pain, headache, rigors, other pain, chills, and fever. Nonspecific symptoms may be included [13].

Mixed-type reactions include both symptoms of type-1 and cytokine release. Nonspecific symptoms may be included [13].

Either-type reactions include only nonspecific symptoms.

Nonspecific symptoms can be seen in all reaction phenotypes [13]. Nonspecific symptoms are flushing, warmth, erythema, unspecific rashes, dyspnea, oxygen desaturation, chest tightness, tachycardia, presyncope, syncope, hypertension, hypotension, nausea, vomiting, abdominal pain, diarrhea, bloating, reflux, numbness, weakness, seizures, unusual taste, and diaphoresis [13].

The RCUH classification of drug hypersensitivity reactions [22] was used for evaluation of reaction severity (Table 1).

**Table 1:** The RCUH classification of drug hypersensitivity to chemotherapeutics and biological agents (22).

Severity grade of DHR	Time to onset of the DHR	Symptoms suggestive of mast cell-mediated reactions	Symptoms suggestive of cytokine release	Common symptoms to both reaction types
**Grade 1: Mild reaction**	Not defined	Pruritus, local urticarial or angioedema	Fever/chills (<38°C), mild Back pain	Erythema
**Grade 2: Moderate reaction** *If a grade 2 symptom is present, any grade 1 symptoms are included*	Slow onset > 15 min	Generalized urticarial and/or angioedema, coryzal symptoms, irritative cough, throat tightness	Severe back pain, chest pain, fever (>38°C)	Dyspnea (SpO_2_ > 92%), nausea, abdominal pain
**Grade 3: Severe reaction** *If there is rapid onset of a grade 2 symptom, it is defined as a grade 3 reaction.* *If a grade 3 symptom is present, any grade 1–2 symptoms are included*	Rapid onset <15 min of grade 2 symptoms or a grade 3 symptom at a not defined time point	Grade 2 symptoms Throat tightness combined with dysphagia, dysphonia or stridor, wheezing	Grade 2 symptoms	Grade 2 symptoms Chest tightness, vomiting, diarrhea, dyspnea (SpO_2_<92%), diaphoresis, dizziness, hypertension
**Grade 4: Anaphylactic shock** *If a grade 4 symptom is present, any grade 1–3 symptoms are included*	Immediate onset or rapid progression			Hypotension, cyanosis, sense of impending doom, faintness, loss of sphincter control, cardiovascular, or respiratory arrest

DHR: Drug hypersensitivity reaction.

### Defining treatment discontinuation

Treatment discontinuation was defined as treatments discontinued due to IDHRs, either after the index reaction or after a failed re-treatment. Treatments discontinued for any other reasons were not included in this group.

### Statistical analysis

The period prevalence with corresponding 95% confidence interval was calculated for IDHRs in total, as well as for different cancer types and for different antineoplastic drugs. We used a chi-squared test for categorical data to test for trend and differences between independent groups. We used the Wilcoxon signed-ranks test for ordinal data to test for differences between matched samples. We used the Kruskal-Wallis test and the Wilcoxon-Mann-Whitney test for ordinal data to test for trend and differences between independent groups. *P* < 0.05 were considered statistically significant. All statistics were performed using StataNow 18 BE (StataCorp, College Station, TX, USA).

### Ethics

This study was approved by the Ethics Committees of Southern Denmark (project-ID S-20220004) and the Danish Data Protection Agency (Journal 22/9106). Written informed consent was obtained from all participants.

## Results

During 17 months, 126 patients were included. The median age was 63 years (range 28–87), 80 (63%) were females, 45 (36%) had an atopic disease, and 18 (15%) had a verified or suspected drug allergy to a nonrelated drug. The distribution of IDHRs within cancer types and antineoplastic drugs is shown in Table 2.

**Table 2:** Prevalence of immediate drug hypersensitivity reactions in patients receiving antineoplastic drug therapy.

Cancer types and drugs	No. of patients with IDHRs/percentage of IDHRs within each cancer type and drug		No. of patients treated during the inclusion period within each cancer type and drug†	Prevalence 95% CI‡
All patients§	124		4994	2.5 [2.1–2.9]
Type of cancer in 124 individual patients¶				
Oncologic§	91	73%	3752	2.4 [1.9–2.9]
Gynecologic§	32	26%	378	8.5 [5.7–11.3]
Breast	14	11%	416	3.4 [1.6–5.1]
Gastrointestinal	22	18%	1105	2.0 [1.2–2.8]
Urologic/prostate	12	10%	600	2.0 [0.9–3.1]
Skin/melanoma	7	6%	395	1.8 [0.5–3.1]
Pulmonologic	4	3%	604	0.7
Hematologic	33	27%	1242	2.7 [1.8–3.6]
Lymphomas/chronic lymphatic leukemia	21	17%	589	3.6 [2.1–5.1]
Myeloma/Waldenstrom	12	10%	434	2.8 [1.2–4.3]
Culprit drugs in 126 index immediate drug hypersensitivity reaction¶				
Platinum salts	23	18%	1762	1.3 [0.8–1.8]
Carboplatin	12	10%	916	1.3 [0.6–2.0]
Cisplatin	3	2%	281	1.1
Oxaliplatin	8	6%	565	1.4 [0.4–2.4]
Taxanes	31	25%	960	3.2 [2.1–4.3]
Abraxane	1	1%	81	1.2
Docetaxel	6	5%	432	1.4 [0.3–2.5]
Paclitaxel	24	19%	447	5.4 [3.3–7.5]
Other chemotherapeutics	26	21%		
Liposomal doxorubicin	8	6%	98	8.2 [2.7–13.6]
Bendamustin	10	8%	321	3.1 [1.2–5.0]
Etoposid	3	2%	177	1.7
Gemcitabin	3	2%	467	0.6
Irinotecan	1	1%	283	0.4
Pemetrexed	1	1%	113	0.9
Checkpoint inhibitors	22	17%	1138	1.9 [1.1–2.7]
Avelumab	3	2%	40	7.5
Cemiplimab	2	2%	28	7.2
Ipilimumab	2	2%	142	1.4
Nivolumab	13	10%	259	5.0 [2.4–7.7]
Pembrolizumab	2	2%	473	0.4
*Other* biologics	24	19%	1386	1.7 [1.0–2.4]
Cetuximab	3	2%	77	3.9
Carfilzomib	1	1%	43	2.3
Daratumumab	2	2%	154	1.3
Obinutuxumab	2	2%	35	5.8
Rituximab	16	13%	366	4.4 [2.3–6.5]

IDHR: Immediate drug hypersensitivity reaction.

† Patients > 18 years receiving intravenous and/or subcutaneous antineoplastic drug therapy at the oncology or hematology outpatient clinic at OUH. Patients often received more than one antineoplastic drugs in combination.

‡ A 95%CI was estimated if there were more than 5 IDHRs in the group.

§ Two patients had two IDHRs to different drugs at different times during the inclusion period.

¶ Cancer types and specific drugs are not included in this table if no IDHRs were seen within the cancer type/drug.

A total of 4994 unique patients received medical antineoplastic drug therapy with an intravenous and/or a subcutaneous drug formulation at the outpatient infusion centers at OUH during the inclusion period. Patients with oncologic cancers accounted for 3752 (75%; Table 2).

### Prevalence of IDHRs

Of 126 patients with IDHRs, two patients were counted twice as they experienced IDHRs with two unrelated drugs at different times during the inclusion period. The 124 unique patients correspond to 2.5% of patients receiving antineoplastic drug therapy (Table 2). The prevalence was 2.4% for patients with oncologic cancers and 2.7% for patients with hematologic cancers. The highest prevalence was found in patients with gynecologic cancers (9%).

For drugs used in at least 250 patients, the prevalence of patients with IDHRs for selected drugs was: paclitaxel 5%, nivolumab 5%, rituximab 4%, bendamustine 3%, oxaliplatin 1%, docetaxel 1%, and carboplatin 1% (Table 2). For drugs used in less than 250 patients, the prevalence of patients with IDHRs for selected drugs was: liposomal doxorubicin 8%, avelumab 8%, cemiplimab 7%, and obinutuzumab 6%.

In a subgroup of patients with gynecologic cancers, the prevalence of IDHRs was 4% (11/269; 95%CI [1.7; 6.5]) among patients treated with carboplatin and 10% (13/135; 95%CI [4.7; 14.6]) among patients treated with paclitaxel.

### Patterns of index immediate drug hypersensitivity reactions

**Figure 1:**
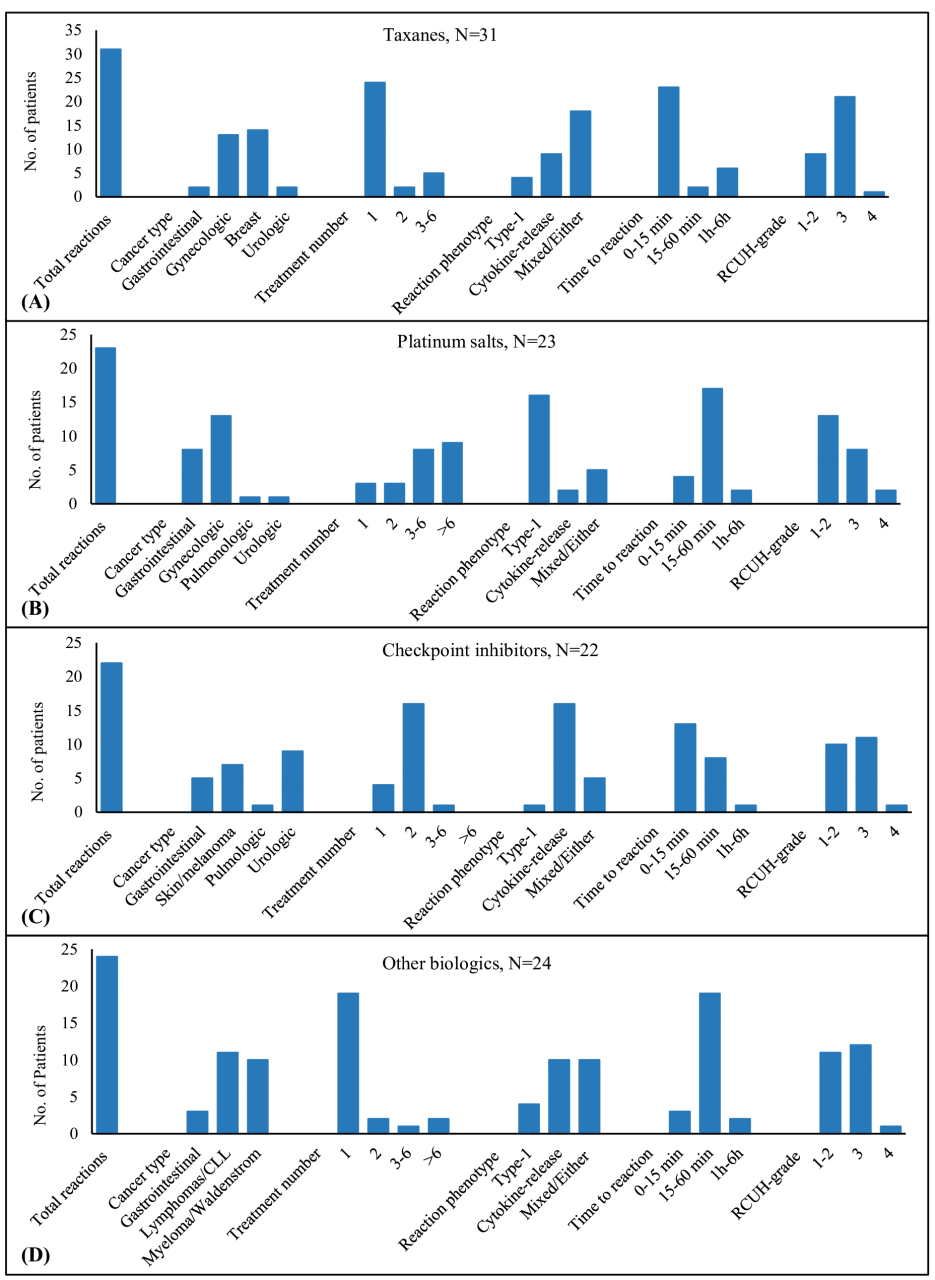
Characteristics of immediate drug hypersensitivity reactions (IDHRs) observed within the four major drug classes. The figure includes information on cancer types for 100 patients reacting to a drug belonging to one of the major drug classes and characteristic of IDHRs observed within the drug classes. The four drug classes are: (A) taxanes, (b) platinum salts, (c) checkpoint inhibitors, and (d) other biologics.

Of 126 index IDHRs, 31 (25%) were elicited by taxanes, 23 (18%) by platinum salts, 22 (17%) by checkpoint inhibitors, and 24 (19%) by *other* biologics. Patterns of IDHRs are shown in relation to drug classes in Figure 1. The distribution of IDHRs between cancer types reflects the use of specific drugs.

With taxanes and *other* biologics, 77% (24/31) and 79% (19/24) of index IDHRs occurred during the first treatment, and the median treatment number was 1 (range 1–6) and 1 (range 1–10), respectively. With checkpoint inhibitors, 73% (16/22) of IDHRs occurred during the second treatment with a median treatment number of 2 (range 1–7). With platinum salts, 74% (17/23) of IDHRs occurred during the fifth treatment or later with a median treatment number of 6 (range 1–15).The difference in median treatment number was significant between each drug class (*p* < 0.001) except for taxanes compared to *other* biologics.

There was a significantly higher proportion of type-1 reactions within platinum salts, 70% (16/23) compared to each of the other drug classes (*p* < 0.001). With checkpoint inhibitors and *other* biologics, cytokine-release was the most common reaction phenotype accounting for 73% (16/22) and 42% (10/24) of IDHRs, respectively. With taxanes, the most common reaction phenotype was the ‘either-type’, accounting for 40% (12/31) of IDHRs. For differences in time to reaction and severity of reactions between drug classes, refer to Figure 1.

### Overview of re-treatments and treatment discontinuations

**Figure 2:**
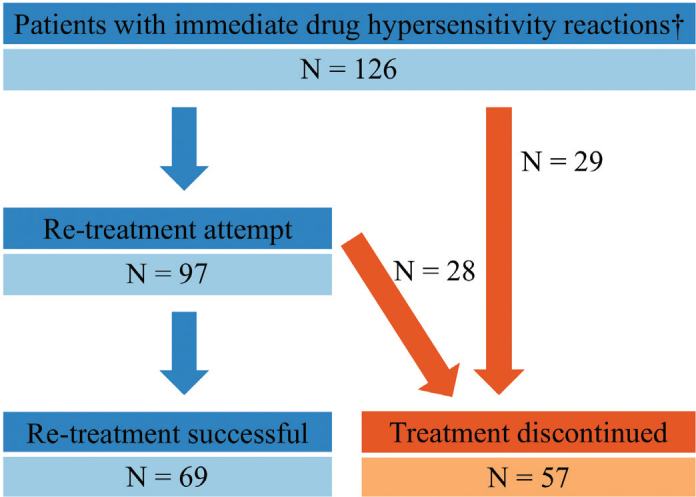
Flowchart of patients with immediate drug hypersensitivity reactions (IDHRs), re-treatment attempts, and outcome of re-treatment. †Two patients are counted twice as reacting to two different drugs at different times during the inclusion period.

Of 126 patients with IDHRs, the antineoplastic treatment was discontinued in 29 (23%) without attempting re-treatment. Of 97 re-treated patients, 28 (29%) had a second IDHR that led to treatment discontinuation. In total, 57 patients discontinued treatment (Figure 2).

One patient discontinued treatment with two unrelated drugs at different times during the inclusion period and was counted twice. The 56 unique patients discontinuing treatment correspond to 1.1% (56/4994, 95%CI [0.8; 1.4]) of patients receiving antineoplastic drug therapy. Of patients with hematologic cancers, 0.7% (9/1242, 95%CI [0.3; 1.2]) discontinued treatment, and of patients with oncologic cancers, 1.3% (47/3752, 95%CI [0.9; 1.6]) discontinued treatment. In the subgroup of patients with gynecologic cancers, it was 7.4% (28/378, 95%CI [4.8; 10.0]).

### Treatments discontinued after the index IDHR

Of patients with IDHRs, 30% (28/93) of patients with oncologic cancers discontinued treatment and 3% (1/33) of patients with hematologic cancers discontinued treatment (Table 3). The difference was significant (*p* < 0.001). Of patients with IDHRs and gynecologic cancers, 62% (21/34) discontinued treatment, and this proportion was significantly higher compared to the other oncologic cancer types combined (*p* < 0.001). When treatments with taxanes, liposomal doxorubicin, and platinum salts were discontinued, the indication for treatment was gynecologic cancer in 8/9, 6/6, and 6/8, respectively.

**Table 3:** Re-treatment and outcome in relation to cancer type, drug class, reaction phenotype, and reaction severity for patients with immediate drug hypersensitivity reactions (IDHR) to antineoplastic drugs.

Characteristics of patients	Index IDHRs			Re-treatment		
	Patients with IDHRs	Patients who discontinue treatment after index IDHR		Re-treated Patients	Patients who discontinue treatment after a failed re-treatment attempt	
	Number	Proportion	Percentage†	Number	Proportion	Percentage†
Total	126	29/126	23%	97	28/97	29%
Cancer type						
Oncologic	93	28/93	30%	65	20/65	31%
Gastrointestinal	22	6/22	27%	16	2/16	13 %
Gynecologic	34	21/34	62%	13	8/13	62%
Breast	14	1/14	7%	13	1/13	8%
Skin/melanoma	7	0/7		7	2/7	
Pulmonologic	4	0/4		4	1/4	
Urologic/prostatae	12	0/12	0%	12	6/12	50%
Hematologic	33	1/33	3%	32	8/32	25%
Lymphomas/CLL	21	1/21	5%	20	3/20	15%
Myeloma/Waldenstrom	12	0/12	0%	12	5/12	42%
Drug						
Platinum salts	23	8/23	35%	15	8/15	53%
Taxanes	31	9/31	29%	22	4/22	18%
Liposomal doxorubicin	8	6/8		2	1/2	
Bendamustine	10	0/10	0%	10	4/10	40%
Other chemotherapeutics	8	1/8		7	0/7	
Checkpoint inhibitors	22	5/22	23%	17	7/17	41%
Other biologics	24	0/24	0%	24	4/24	17%
Reaction phenotype						
Type 1	27	11/27	41%	16	8/16	50%
CRR	49	8/49	16%	41	10/41	24%
Mixed	19	5/19	26%	14	4/14	29%
Either	31	5/31	16%	26	6/26	23%
RCUH-Grade: Index IDHR						
1–2	53	6/53	11%	47	14/47	30%
3	63	16/63	25%	47	14/47	30%
4	10	7/10	70%	3	0/3	

IDHR: Immediate drug hypersensitivity reaction.

† Percentage is given, if there is at least 10 patients in the group.

The proportion of patients discontinuing treatment was higher among patients with severe/anaphylactic IDHRs (RCUH-grade 3–4) compared to mild/moderate IDHR (*p* < 0.01) (Table 3). However, only 25% (16/63) of treatments were discontinued after a severe (RCUH-grade 3) index IDHR, and re-treatment was attempted in three patients after an anaphylactic reaction (RCUH-grade 4).

### Re-treatment strategies

**Figure 3:**
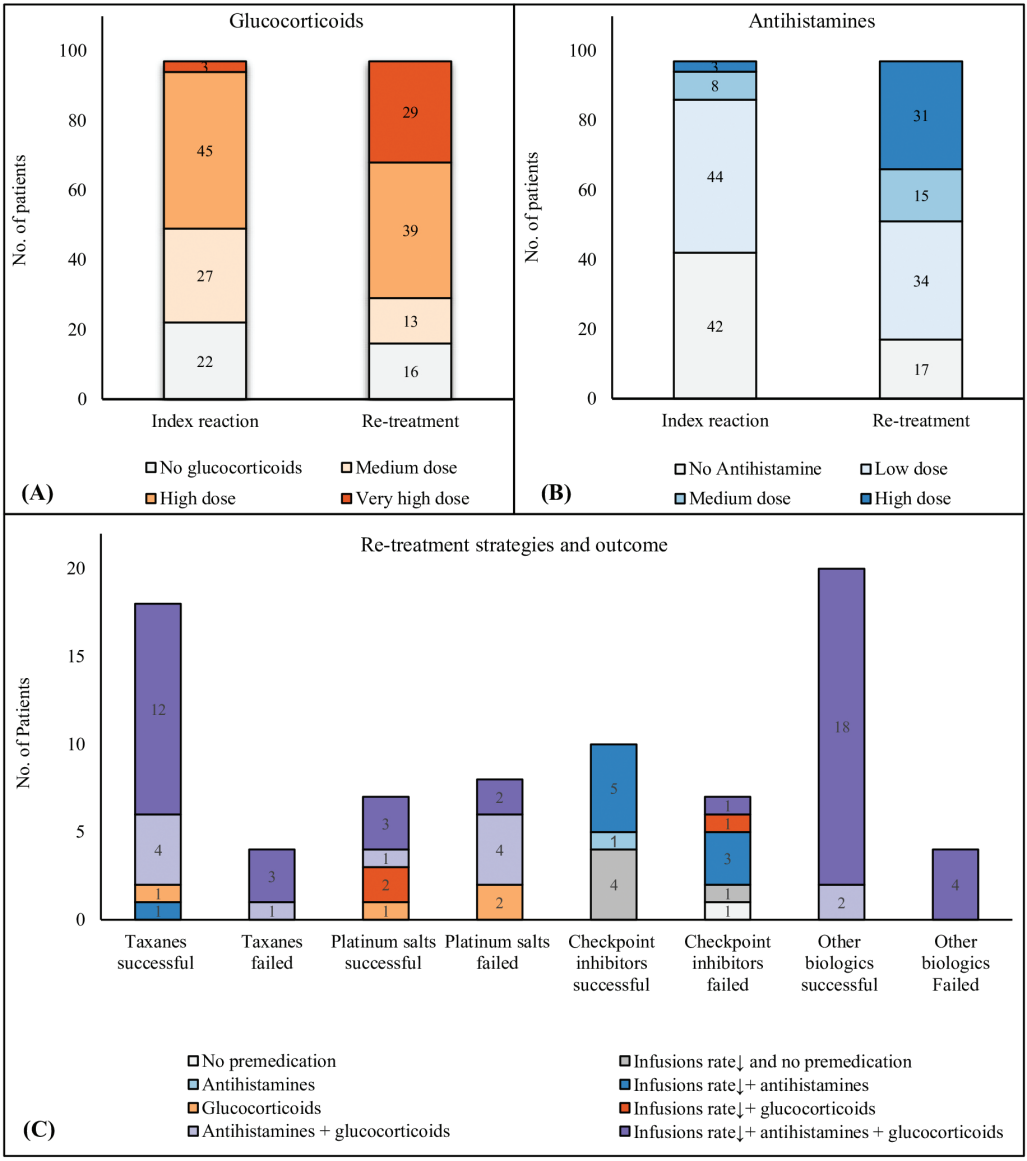
Use of premedication and re-treatment strategies. (A–B) Overview of the level of premedication used as standard prior to the index reaction and the level of premedication used prior to re-treatment in 97 re-treated patients. (A) Glucocorticoids: Equivalent prednisolone doses: Medium dose: 50 mg, High dose: 100 mg, Very high dose > 100 mg. (B) Antihistamines: Low dose: Oral antihistamine in recommended dose, Medium dose: 1 mg clemastine, High dose > 1 mg clemastine. (C) Re-treatment strategies and outcome (successful re-treatment/failed re-treatment) observed within the four major drug classes, including 78 patients (taxanes n = 22, platinum salts n = 15, checkpoint inhibitors n = 17, other biologics n = 24). Re-treatment strategies included different combinations of antihistamines (used in different dosages), glucocorticoids (used in different dosages), and decreased infusion rate (a decreased infusion rate was equivalent to 50% of a standard infusion rate).

Of 97 re-treated patients, 81% (79/97) had received premedication prior to the index IDHR following standards at cancer departments. A combination of glucocorticoids and antihistamines was used in 53% (51/97) (doses are indicated in Figure 3A–B). Patients treated with platinum salts (*n* = 15) all received glucocorticoids as antiemetic. Of patients treated with taxanes and *other* biologics, 91% (20/22) and 92% (22/24) received a combination of glucocorticoids and antihistamines, respectively. Of patients treated with checkpoint inhibitors, 88% (15/17) received no premedication.

At re-treatment, strategies to avoid new IDHRs were to increase premedication (antihistamines and/or glucocorticoids) in 22% (21/97), decrease infusion rate in 25% (24/97) or both in 44% (43/97). Premedication was used in 94% (91/97) and 72% (70/97) received a combination of glucocorticoids and antihistamines (doses are indicated in Figure 3A–B). In total, 69% (67/97) had a decreased infusion rate during retreatment, and patients received significantly more premedication compared to standard premedication applied prior to the index IDHR (*p* < 0.0001).

As illustrated in Figure 3C, patients received different combinations of premedication and decreased/normal infusion rate during re-treatment, both between drug classes and within the same drug class.

### Treatments discontinued after failed re-treatments

The outcome of re-treatment in relation to re-treatment strategies is shown in Figure 3C. Different combinations of antihistamines/glucocorticoids and decreased infusion rate ensured a successful re-treatment within all drug classes. However, failed re-treatments were also observed within all drug classes despite using a combination of glucocorticoids, antihistamines, and decreased infusion rate. Of 28 IDHRs observed during re-retreatments, 68% (19/28) were severe (RCUH-grade 3), and 14% (4/28) were anaphylactic shock (RCUH-grade 4). Of patients with anaphylactic shock, index IDHRs were of type-1 phenotype, mild to moderate in severity, and occurred during treatments with previously tolerated drugs. The culprit drugs were carboplatin (*n* = 2), paclitaxel (*n* = 1), and rituximab (*n* = 1).

The proportion of re-treated patients discontinuing treatment did not differ significantly between patients with oncologic and hematologic cancers. Among patients with gynecologic cancers, the proportion of re-treated patients discontinuing treatment was 62% (8/13), which was significantly higher compared to the other oncologic cancer types combined (*p* < 0.01). In hematologic cancers, no significant difference was found between cancer types.

There was a significant difference in the proportion of re-treated patients discontinuing treatment between chemotherapeutic drug classes. This difference was identified to be between platinum salts 53% (8/15) and taxanes 18% (4/22) (*p* < 0.05). There was no difference in the proportion of patients discontinuing treatment between biologic drug classes. The highest proportion was observed with checkpoint inhibitors 41% (7/17).

There was no significant difference in the proportion of patients discontinuing treatment between reaction phenotypes. The highest proportion of 50% (8/15) was observed in type-1 reactions. The proportion of treatment discontinuations did not differ with the severity of the index reaction.

## Discussion

In this prospective observational study, 126 patients experienced IDHRs over 17 months, corresponding to 2.5% of patients receiving antineoplastic drug therapy. Re-treatment, using increased premedication and/or decreased infusion rate, was attempted in 97 patients and tolerated by 69. However, 57 out of 126 patients (45%) discontinued treatment. This corresponds to 1.1% of patients receiving antineoplastic drug therapy. Of patients with gynecologic cancer, 9% experienced IDHRs and 7.4% discontinued treatment.

Approximately 250 patients per year in Denmark will discontinue treatment with an antineoplastic drug because of IDHRs. This is based on the expected number of patients receiving antineoplastic drug therapy in Denmark [23, 24], and that 1.1% will discontinue a drug treatment due to IDHRs. Patients discontinuing treatment in this study do not differ from patients offered RDD in specialized allergy units in Southern Europe and the US [1, 2]. If Danish patients were offered RDD, they might be able to continue treatments that are currently being discontinued [1, 2]. RDD may be relevant for patients who have failed re-treatments, patients with type-1 reactions (especially observed in – but not limited to - treatments with platinum salts) and when the severity of IDHRs prevents cancer departments from re-treating patients themselves.

No similar studies report the overall prevalence of IDHRs in antineoplastic drug therapy. For specific drugs, the prevalence of IDHRs reported here is comparable to other studies [3, 7–10, 21, 25, 26]. Consistent with these same studies, we found that IDHRs elicited by taxanes and *other* biologics mainly occurred in the first treatment, suggesting a nonspecific immune response. In addition, IDHRs elicited by platinum salts mainly have a type-1 phenotype and occur after multiple exposures, suggesting a specific immune response. With checkpoint inhibitors, IDHRs mainly occurred in the second treatment. This is not reported elsewhere, and the reason is unclear.

The high prevalence of IDHRs in gynecologic cancers reflects the use of antineoplastic drugs. With platinum salts, the risk of IDHRs increases with the total number of treatments [2]. In gynecologic cancers, carboplatin is used in multiple lines of therapy. Paclitaxel and liposomal doxorubicin are other drugs used for gynecologic cancers that are known to cause IDHRs [3].

The highest proportion of patients discontinuing treatment after the index IDHR was found within gynecologic cancers. Compared to patients with breast cancer, treatments with paclitaxel were more frequently discontinued. One explanation could be that the dosage of paclitaxel differs between cancer types, as patients with gynecologic cancer were treated with paclitaxel at 175 mg/m^2^ every 3 weeks in combination with carboplatin, while patients with breast cancer received weekly paclitaxel at 80 mg/m^2^. Furthermore, the proportion of patients discontinuing treatment after failed re-treatments was highest within gynecologic cancers, which may influence the decision to attempt re-treatment in the first place.

Of 97 re-treated patients, 69 tolerated re-treatment, suggesting that increased premedication and/or decreased infusion rate can be important tools to enable continued treatment at cancer departments. However, it is unclear to what extent premedication and decreased infusion rate contribute to re-treatment success, as different combinations of glucocorticoids, antihistamines, and infusion rates were observed in both successful and unsuccessful re-treatments. Information of optimal re-treatment strategies is lacking [27].

Re-treatment is generally reported to be difficult with platinum salts and successful with taxanes, *other* biologics, and checkpoint inhibitors [4, 5, 17, 19, 21]. Our results for taxanes, platinum salts, and *other* biologics are consistent with this, while with checkpoint inhibitors it was not, as 41% (7/17) of re-treatments were unsuccessful.

Severity of index IDHRs played a role in which patient were re-treated. However, re-treatment success was similar between patients with mild/moderate (RCUH-grade 1–2) and severe/anaphylactic (RCUH-grade 3–4) IDHRs. In fact, anaphylactic shocks were observed during re-treatments following mild/moderate type-1 IDHRs elicited by drugs previously tolerated by the patient. This suggests that the clinical picture of classic allergic (type-1) symptoms occurring after a period of possible sensitization should prompt health care professional to be extra cautious when considering re-treating patients.

## Strengths and limitations

This study was conducted in one of the largest hospitals in Denmark, treating adults with all cancer diseases except a few rare types. We cannot be assured that all eligible patients were included. Patients were only included from the outpatient infusion center, where 92% of patients are treated. Patients with hematologic cancers receiving the most intensive treatments are treated in the inpatient ward, which could be associated with a higher risk of IDHRs [28]. The true prevalence of IDHRs may thus be higher than reported here.

We categorized IDHRs into phenotypes based on symptoms alone, which can be easily obtained in all settings. The proportion of patients with type-1 *reactions* in this study is smaller than in others [12]. This is probably due to the inclusion of the reaction phenotype ‘either-type’, which includes IDHRs with only nonspecific symptoms such as flushing and dyspnea [13]. Type-1 reactions include at least one symptom suggestive of mast cell degranulation, typical of (but not limited to) IgE-mediated allergic reactions [29]. Phenotyping of IDHRs is not perfect, but it seems important to distinguish between type-1 reactions and other reaction phenotypes.

The prospective design of this study allowed us to interview and observe patients while the IDHR was still ongoing or immediately after and to retrieve information from the nurse performing the treatment. Information on subjective and objective symptoms, premedication, and infusion rates is thus accurate.

We observed the management of IDHRs in antineoplastic drug therapy without interfering with normal procedures. This provides a unique picture of how IDHRs are managed in a setting where allergists are not involved in the evaluation of patients with IDHRs in antineoplastic drug therapy and where RDD is not implemented as standard treatment options.

## Conclusion

In this study, we found that 2.5% of patients in antineoplastic drug therapy experience IDHRs and that 1.1% discontinue treatment with the culprit drug due to such reactions. Patients with gynecologic cancers had a particularly high risk.

Re-treatment using decreased infusion rate and/or increased premedication can be important tools to enable patients to continue treatment at the cancer departments. However, re-treatment is not always advisable and not always successful. In this study, 45% (57/126) of patients with IDHRs discontinued treatment with the culprit drug. It seems reasonable to consider whether an allergy work-up including RDD could be beneficial in these situations.

## Author contribution

THR and CBJ conceived the study design. THR observed and interviewed patients and healthcare professionals and extracted data from medical files. CGM and THR performed statistical analysis. All authors were involved in data interpretation. THR wrote the manuscript. All authors reviewed and approved the final manuscript.

## Data Availability

Data can be made available upon request to the corresponding author.

## References

[1] Alvarez-Cuesta E, Madrigal-Burgaleta R, Broyles AD, Cuesta-Herranz J, Guzman-Melendez MA, Maciag MC, et al. Standards for practical intravenous rapid drug desensitization & delabeling: a WAO committee statement. World Allergy Organ J. 2022;15(6):100640. 10.1016/j.waojou.2022.100640 35694005 PMC9163606

[2] Pagani M, Bavbek S, Alvarez-Cuesta E, Berna Dursun A, Bonadonna P, Castells M, et al. Hypersensitivity reactions to chemotherapy: an EAACI Position Paper. Allergy. 2021;77(2):388–403. 10.22541/au.161726282.21370610/v1 34587281

[3] Pagani M. The complex clinical picture of presumably allergic side effects to cytostatic drugs: symptoms, pathomechanism, reexposure, and desensitization. Med Clin North Am. 2010;94(4):835–52, xiii. 10.1016/j.mcna.2010.03.002 20609866

[4] Picard M, Castells MC. Re-visiting hypersensitivity reactions to taxanes: a comprehensive review. Clin Rev Allergy Immunol. 2015;49(2):177–91. 10.1007/s12016-014-8416-0 24740483

[5] Navo M, Kunthur A, Badell ML, Coffer LW, 2nd, Markman M, Brown J, et al. Evaluation of the incidence of carboplatin hypersensitivity reactions in cancer patients. Gynecol Oncol. 2006;103(2):608–13. 10.1016/j.ygyno.2006.04.002 16797060

[6] Rogers BB, Cuddahy T, Briscella C, Ross N, Olszanski AJ, Denlinger CS. Oxaliplatin: Detection and management of hypersensitivity reactions. Clin J Oncol Nurs. 2019;23(1):68–75. 10.1188/19.CJON.68-75 30682002

[7] Bavbek S, Pagani M, Alvarez-Cuesta E, Castells M, Dursun AB, Hamadi S, et al. Hypersensitivity reactions to biologicals: an EAACI position paper. Allergy. 2021;77(1):39–54. 10.22541/au.161718382.20133527/v1 34157134

[8] Hiraizumi K, Honda C, Watanabe A, Nakao T, Midorikawa S, Abe H, et al. Safety of nivolumab monotherapy in five cancer types: pooled analysis of post-marketing surveillance in Japan. Int J Clin Oncol. 2024;29(7):932–43. 10.1007/s10147-024-02515-1 38844668 PMC11196337

[9] Yamamoto N, Nakanishi Y, Gemma A, Nakagawa K, Sakamoto T, Akamatsu A, et al. Real-world safety of nivolumab in patients with non-small-cell lung cancer in Japan: postmarketing surveillance. Cancer Sci. 2021;112(11):4692–701. 10.1111/cas.15117 34431585 PMC8586674

[10] Momtaz P, Park V, Panageas KS, Postow MA, Callahan M, Wolchok JD, et al. Safety of infusing ipilimumab over 30 minutes. J Clin Oncol. 2015;33(30):3454–8. 10.1200/JCO.2015.61.0030 26124475 PMC5087314

[11] Jakubovic BD, Vecillas LL, Jimenez-Rodriguez TW, Sanchez-Sanchez S, Castells M. Drug hypersensitivity in the fast lane: what clinicians should know about phenotypes, endotypes, and biomarkers. Ann Allergy Asthma Immunol. 2020;124(6):566–72. 10.1016/j.anai.2020.04.005 32302769

[12] Isabwe GAC, Garcia Neuer M, De Las Vecillas Sanchez L, Lynch DM, Marquis K, Castells M. Hypersensitivity reactions to therapeutic monoclonal antibodies: phenotypes and endotypes. J Allergy Clin Immunol. 2018;142(1):159–70.e2. 10.1016/j.jaci.2018.02.018 29518427

[13] Silver J, Garcia-Neuer M, Lynch DM, Pasaoglu G, Sloane DE, Castells M. Endophenotyping oxaliplatin hypersensitivity: personalizing desensitization to the atypical platin. J Allergy Clin Immunol Pract. 2020;8(5):1668–80.e2. 10.1016/j.jaip.2020.02.013 32112926

[14] O’Cearbhaill R, Zhou Q, Iasonos A, Hensley ML, Tew WP, Aghajanian C, et al. The prophylactic conversion to an extended infusion schedule and use of premedication to prevent hypersensitivity reactions in ovarian cancer patients during carboplatin retreatment. Gynecol Oncol. 2010;116(3):326–31. 10.1016/j.ygyno.2009.10.070 19944454 PMC4369374

[15] Lenz HJ. Management and preparedness for infusion and hypersensitivity reactions. Oncologist. 2007;12(5):601–9. 10.1634/theoncologist.12-5-601 17522249

[16] Markman M, Kennedy A, Webster K, Kulp B, Peterson G, Belinson J. Paclitaxel-associated hypersensitivity reactions: experience of the gynecologic oncology program of the Cleveland Clinic Cancer Center. J Clin Oncol. 2000;18(1):102–5. 10.1200/JCO.2000.18.1.102 10623699

[17] Banerji A, Lax T, Guyer A, Hurwitz S, Camargo CA, Jr., Long AA. Management of hypersensitivity reactions to Carboplatin and Paclitaxel in an outpatient oncology infusion center: a 5-year review. J Allergy Clin Immunol Pract. 2014;2(4):428–33. 10.1016/j.jaip.2014.04.010 25017531

[18] Broyles AD, Banerji A, Barmettler S, Biggs CM, Blumenthal K, Brennan PJ, et al. Practical guidance for the evaluation and management of drug hypersensitivity: specific drugs. J Allergy Clin Immunol Pract. 2020;8(9s):S16–S116. 10.1016/j.jaip.2020.08.002 33039007

[19] Park BC, Stone CA Jr., Dewan AK, Johnson DB. Hypersensitivity reactions and immune-related adverse events to immune checkpoint inhibitors: approaches, mechanisms, and models. Immunol Allergy Clin North Am. 2022;42(2):285–305. 10.1016/j.iac.2021.12.006 35469619

[20] Markman M, Kennedy A, Webster K, Elson P, Peterson G, Kulp B, et al. Clinical features of hypersensitivity reactions to carboplatin. J Clin Oncol. 1999;17(4):1141. 10.1200/JCO.1999.17.4.1141 10561172

[21] Tsao LR, Young FD, Otani IM, Castells MC. Hypersensitivity reactions to platinum agents and taxanes. Clin Rev Allergy Immunol. 2022;62(3):432–48. 10.1007/s12016-021-08877-y 34338975 PMC9156473

[22] Madrigal-Burgaleta R, Bernal-Rubio L, Berges-Gimeno MP, Carpio-Escalona LV, Gehlhaar P, Alvarez-Cuesta E. A large single-hospital experience using drug provocation testing and rapid drug desensitization in hypersensitivity to antineoplastic and biological agents. J Allergy Clin Immunol Pract. 2019;7(2):618–32. 10.1016/j.jaip.2018.07.031 30098410

[23] Danish Health Authorities. Specialty guideline for clinical oncology 2023. [Citation date: 10.01.2025] Available from: https://www.sst.dk/-/media/Viden/Specialplaner/Specialeplan-for-klinisk-onkologi/Specialevejledning-for-Klinisk-onkologi-den-25-juli-2023.ashx?sc_lang=da&hash=6DCD53183F1ABB1B488D9053EA6B6CFF

[24] Danish Health Authorities. Specialty guideline for clinical hematology 2023. [Citation date: 10.01.2025] Available from: https://www.sst.dk/-/media/Viden/Specialplaner/Specialeplan-for-intern-medicin-haematologi/Specialevejledning-for-Intern-medicin-Haematologi-den-15-marts-2023.ashx

[25] Cheung EM, Edenfield WJ, Mattar B, Anthony SP, Mutch PJ, Chanas B, et al. Safety and pharmacokinetics of bendamustine rapid-infusion formulation. J Clin Pharmacol. 2017;57(11):1400–8. 10.1002/jcph.942 28561902

[26] Santos RB, Galvão VR. Monoclonal antibodies hypersensitivity: prevalence and management. Immunol Allergy Clin North Am. 2017;37(4):695–711. 10.1016/j.iac.2017.07.003 28965635

[27] Zwimpfer TA, Bilir E, Gasimli K, Cokan A, Bizzarri N, Razumova Z, et al. Management of patients with hypersensitivity to platinum salts and taxane in gynecological cancers: a cross-sectional study by the European Network of Young Gynaecologic Oncologists (ENYGO). Cancers (Basel). 2024;16(6):1155. 10.3390/cancers16061155 38539490 PMC10969349

[28] Chung CH. Managing premedications and the risk for reactions to infusional monoclonal antibody therapy. Oncologist. 2008;13(6):725–32. 10.1634/theoncologist.2008-0012 18586928

[29] Cardona V, Ansotegui IJ, Ebisawa M, El-Gamal Y, Fernandez Rivas M, Fineman S, et al. World allergy organization anaphylaxis guidance 2020. World Allergy Organ J. 2020;13(10):100472. 10.1016/j.waojou.2020.100472 33204386 PMC7607509

